# Development of a prediction model for acute kidney injury in critically ill patients with advanced colorectal cancer based on white blood cell-related indicators

**DOI:** 10.3389/fonc.2026.1768330

**Published:** 2026-04-27

**Authors:** Lingjie Li, Pu Zhou, YuanJin Guo, Haragakiza Jean Docile, David Fisher, Nguyen Thi Thu Hien, Erkin Musabaev, Vadim V. Shmanai, Yiping Dang, Zhide Zhu, Lei Zhao, Jie Xu

**Affiliations:** 1 China Three Gorges University, Yichang, Hubei, China; 2 Department of Infectious Diseases, Union Hospital, Tongji Medical College, Huazhong University of Science and Technology, Wuhan, Hubei, China; 3 Department of Neurology, Union Hospital, Tongji Medical College, Huazhong University of Science and Technology, Wuhan, Hubei, China; 4 Department of Medical Biosciences, Faculty of Natural Sciences, University of the Western Cape, Cape Town, South Africa; 5 Hai Phong University of Medicine and Pharmacy, Hai Phong, Vietnam; 6 The Research Institute of Virology, Ministry of Health, Tashkent, Uzbekistan; 7 Institute of Physical Organic Chemistry, National Academy of Sciences of Belarus, Minsk, Belarus; 8 Department of Vascular Surgery, Union Hospital, Tongji Medical College, Huazhong University of Science and Technology, Wuhan, China; 9 The First Clinical Medical College, Guangxi University of Chinese Medicine, Nanning, Guangxi, China; 10 Department of Spleen and Stomach Diseases, Yichang Hospital of Chinese Medicine & China Three Gorges University Chinese Medicine Hospital, Yichang, Hubei, China

**Keywords:** acute kidney injury, colorectal cancer, eICU, prediction model, white blood cell

## Abstract

**Background:**

Currently, most prediction models for acute kidney injury (AKI) in patients with colorectal cancer (CRC) are only applicable to postoperative situations, or rely on complex machine learning algorithms. There is currently no simple and feasible AKI prediction model for severely ill patients with advanced CRC.

**Objective:**

This study endeavors to devise and validate a novel clinical risk prediction model utilizing white blood cell (WBC)-related indicators to forecast AKI in critically ill patients with advanced CRC.

**Methods:**

A retrospective cohort study design was employed. The training cohort was derived from the eICU Collaborative Research Database, and the external validation cohort came from the Intensive Care Unit (ICU) of Wuhan Union Hospital. To screen for independent predictors of AKI and develop a new risk score model using multivariable logistic regression analysis. The assessment of model performance was conducted using receiver operating characteristic (ROC) curve analysis and compared with other scoring systems. The associations between different risk groups and survival outcomes as well as WBC counts were visualized using a heatmap. Kaplan-Meier survival analysis was used to compare in-hospital survival rates between the two groups. ROC curve analysis and decision curve analysis (DCA) served to evaluate the model’s predictive ability for in-hospital mortality. Subgroup analysis was applied to assess the consistency of this model across different critically ill cohorts with advanced CRC.

**Results:**

A total of 981 critically ill patients with advanced CRC were included, of whom 185 (24.2%) from the eICU database and 59 (27.2%) from the Wuhan Union Hospital ICU developed AKI. A new risk model was successfully established: 1.271×WNR+0.709×Lg(WER)+0.085×MLR+1.225×Lg(SII). The model showed acceptable discrimination for predicting AKI, with area under the curve (AUC) of 0.746 (95% CI, 0.704-0.788) in the training set and 0.716 (95% CI, 0.633-0.798) in the validation set, and provided good clinical net benefit. For predicting in-hospital mortality, the AUCs were 0.788 (95% CI, 0.736-0.840) and 0.693 (95% CI, 0.559-0.827) in the training and validation sets, respectively.

**Conclusion:**

This study developed a novel prediction model for AKI in critically ill patients with advanced CRC using WBC-related indicators. This model offers a simple, readily implementable tool for AKI risk stratification in critically ill patients with advanced CRC, supporting early clinical intervention and prognostic assessment.

## 1 Introduction

Colorectal cancer (CRC) represents a malignancy characterized by a notably high incidence rate worldwide. The GLOBOCAN 2020 report indicates that CRC ranks as the third most prevalent cancer and holds the second position in terms of mortality rates associated with malignant tumors (1). At the same time, this disease with a high mortality rate shows a trend of becoming younger. Studies have shown that although the incidence of CRC in the elderly has decreased, the incidence in people under 50 years old is increasing, in the United States (2). Moreover, more than 60% of these early-onset CRC patients have developed the disease to an advanced stage (III/IV stage) (2). These epidemiological characteristics indicate that patients with CRC not only face high incidence and high mortality rates, but also have the characteristics of younger onset. We need to continuously explore measures for its effective management.

Among critically ill patients, particularly those diagnosed with malignant neoplasms, acute kidney injury (AKI) is a common complication characterized (3). AKI often progresses to chronic kidney disease (CKD), which may necessitate renal replacement therapy (RRT) in severe cases, thereby elevating both the healthcare burden and mortality risk (4). Previous studies have consistently documented that the mortality rate among cancer patients who develop AKI is significantly elevated. A substantial body of evidence reports a rate as high as 47.4%, which is strikingly higher than that observed in cancer patients without this complication (5). Twenty percent of patients diagnosed with CRC experienced acute kidney injury postoperatively within a week following surgery, according to a cohort study based on the Danish medical database (6). Additionally, patients with AKI experienced a higher mortality rate (10.1%) within 8 to 30 days, with the rate among those without AKI being substantially lower (2.2%) (6).

In recent years, several novel biomarkers have been proposed for early AKI detection, including kidney injury molecule-1 (KIM-1), neutrophil gelatinase-associated lipocalin (NGAL), interleukin-18 (IL-18), serum cystatin C, and urinary exosomal microRNA-21. Compared with traditional indicators such as serum creatinine, these markers generally offer earlier detection—ranging from several hours to up to two days—across various AKI etiologies, including ischemic, toxic, and septic insults (7–12). However, their translation into routine clinical practice, particularly in the Intensive Care Unit (ICU), faces notable barriers. First, the detection of KIM-1, NGAL, IL-18, and urinary exosomal microRNA-21 requires specialized methods such as enzyme-linked immunosorbent assay (ELISA) or polymerase chain reaction (PCR), which are associated with longer turnaround times, higher costs, and limited availability in many clinical settings (7, 8, 10, 12). Second, the levels of NGAL and IL-18 can be influenced by factors beyond kidney injury, such as systemic inflammation, CKD, or malignancy itself, which may compromise specificity and contribute to false-positive results in complex critically ill populations (9, 11). These limitations are particularly relevant for patients with advanced CRC, whose disease course is frequently complicated by tumor-related inflammation, chemotherapy-induced nephrotoxicity, and multi-organ dysfunction. Critically ill patients often face high treatment costs and limited therapeutic options; higher detection costs and longer turnaround times would significantly diminish predictive utility. These markers clearly fail to meet the urgent demand for rapid, economical, and easily accessible tools in the ICU environment.

White blood cells (WBC), or leukocytes, serve as prevalent analytical indicators for assessing immunological functions and inflammatory processes. Key measurements include the neutrophil-to-lymphocyte ratio (NLR), platelet-to-lymphocyte ratio (PLR), monocyte-to-lymphocyte ratio (MLR), and additional similar metrics. Baseline diagnostic evaluations, including standard testing, are routinely administered to individuals upon admission to the hospital for critical illness. A growing body of evidence indicates that disturbances in WBC-associated parameters are strongly linked to an increased risk of AKI and poor prognosis. This is corroborated by findings that the platelet-to-white blood cell ratio (PWR) serves as an independent predictor of AKI among surgical patients (13). Additionally, in the ICU, WBC count demonstrates a distinct U-shaped correlation with the occurrence of AKI and patient mortality (14). Advanced CRC is often accompanied by systemic inflammatory response and immune dysregulation. Evidence suggests that NLR and PLR are important factors for predicting the long-term outcomes of patients with stage III-IV CRC (15). WBC-related indicators are closely related to the disease dynamics, clinical prognosis and treatment response in CRC patients, effectively reflecting the condition of patients with advanced CRC. This study aims to establish and validate a new prediction model for AKI in patients with advanced CRC using WBC-related indicators. It is intended to provide ICU healthcare providers with a convenient and reliable tool for risk stratification, early identification, and tracking management of AKI risk populations among critically ill patients with advanced CRC.

## 2 Research methods and materials

### 2.1 Data sources

This study used data from the eICU Collaborative Research Database (16) and the Wuhan Union Hospital database. The study was conducted in accordance with the Declaration of Helsinki. Ethical approval was obtained from the Ethics Committee of Union Hospital, Tongji Medical College, Huazhong University of Science and Technology (No. 2018-S377). The requirement for written informed consent was waived in accordance with national legislation and institutional requirements.

### 2.2 Study population

A total of 3,453 adult ICU patients with advanced CRC were initially identified from the eICU database, and 691 patients were screened from the ICU database of Wuhan Union Hospital between June 2020 and July 2025 using ICD-9 and ICD-10 diagnostic codes. Data from the eICU database were used as the training cohort, whereas data from the Wuhan Union Hospital ICU served as the external validation cohort.

For both cohorts, patients were excluded if they had repeated ICU admissions, multiple hospitalizations, a hospital stay of less than 48 hours, advanced kidney disease, or incomplete WBC measurements. Ultimately, 764 patients were included in the training cohort and 217 patients in the validation cohort.

**Figure 1:**
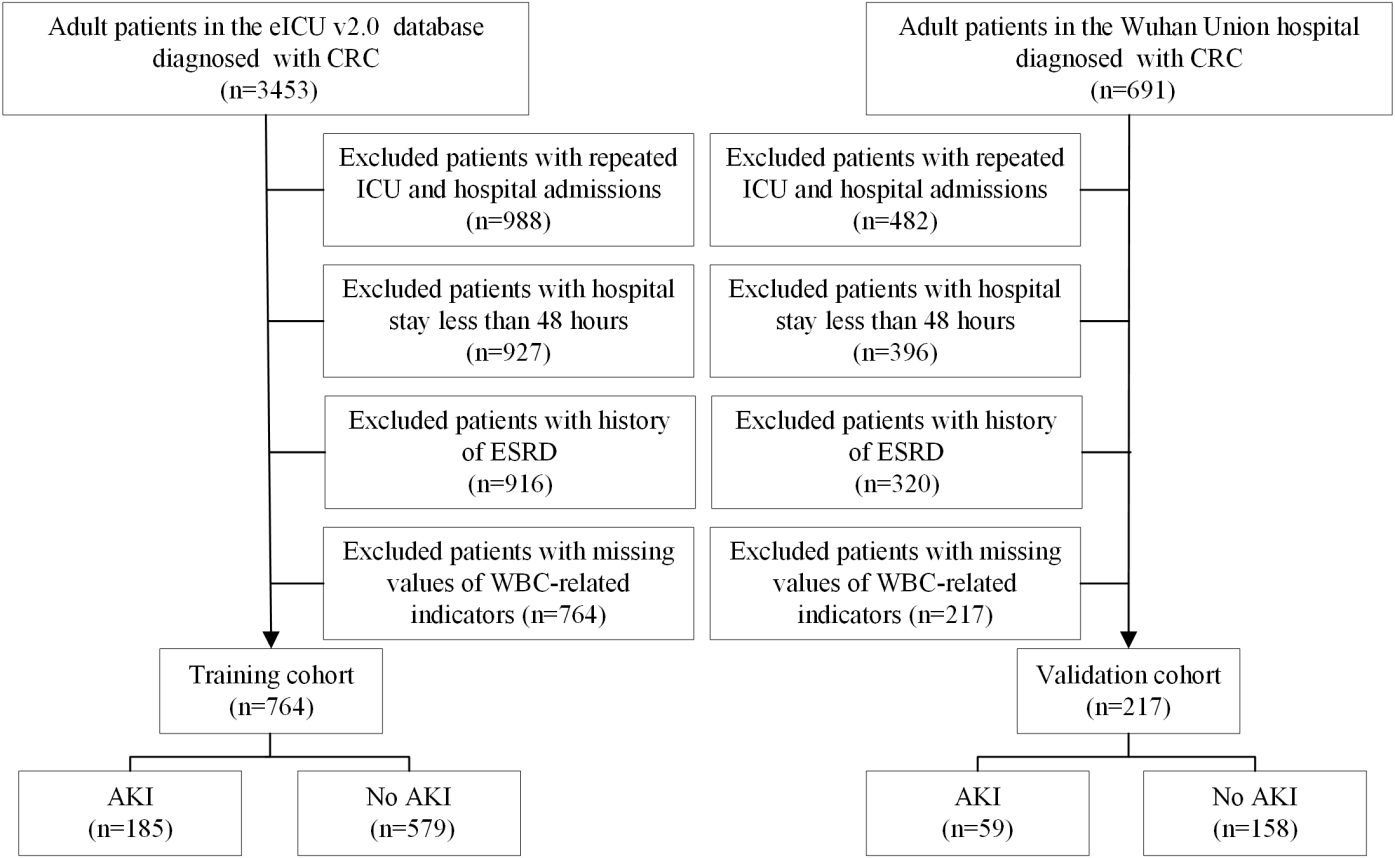
Flowchart of the study.

AKI was defined according to the Kidney Disease: Improving Global Outcomes (KDIGO) criteria as any of the following (17): an increase in serum creatinine of at least 0.3 mg/dL within 48 hours, an increase in serum creatinine to at least 1.5 times baseline within 7 days, or a urine output of less than 0.5 mL/kg/h for 6 hours. Baseline serum creatinine was defined as the lowest value recorded within 48 hours before ICU admission or the first measured value on admission. The study flowchart is shown in **Figure 1**.

### 2.3 Clinical variables and outcome measures

The collected clinical variables included: (1) demographic characteristics, including age (years old), body mass index (BMI, kg/m^2^), and sex; (2) comorbidities, including congestive heart failure, hypertension, diabetes mellitus, and CKD; (3) severity scores, including the Sequential Organ Failure Assessment (SOFA) score, Oxford Acute Severity of Illness Score (OASIS), and Acute Physiology Score III (APS III); (4) laboratory variables, including WBC count (×10^9/L), hemoglobin (HGB, g/dL), platelet count (PL, ×10^9/L), albumin (ALB, g/dL), bilirubin (mmol/L), bicarbonate (mEq/L), blood urea nitrogen (BUN, mg/dL), creatinine (mg/dL), potassium (mmol/L), and sodium (mmol/L); (5) patient clinical outcomes, including length of hospital stay and in-hospital mortality (**Table 1**).

**Table 1:** Baseline characteristics.

Characteristics	Training set (n=764)			Validation set (n=217)		
	No AKI (n=579)	AKI (n=185)	P	No AKI (n=159)	AKI (n=58)	P
Age, years old	69.6 ± 13.5	70.8 ± 13.4	0.308	60.3 ± 13.1	60.6 ± 12.7	0.866
Gender, male, n (%)	262 (45.3)	67 (36.2)	0.031	109 (68.6)	33 (56.9)	0.110
BMI, kg/m2	27.1 ± 6.7	27.5 ± 6.7	0.486	23.9 ± 3.4	23.8 ± 3.3	0.931
Comorbidities, n (%)						
Congestive heart failure	50 (8.6)	25 (13.5)	0.052	47 (29.6)	14 (24.1)	0.432
Hypertension	306 (52.8)	110 (59.5)	0.116	55 (34.6)	19 (32.8)	0.801
Diabetes	135 (23.3)	48 (25.9)	0.466	41 (25.8)	18 (31.0)	0.442
Chronic kidney disease	43 (7.4)	30 (16.2)	<0.001	17 (10.7)	15 (25.9)	0.005
Score system, points						
SOFA	2.9 ± 1.0	5.2 ± 1.5	<0.001	2.4 ± 1.0	4.8 ± 1.5	<0.001
OASIS	22.6 ± 9.7	29.5 ± 10.2	<0.001	19.6 ± 5.7	23.6 ± 6.1	<0.001
APSIII	41.8 ± 16.0	50.6 ± 19.6	<0.001	30.1 ± 7.1	39.9 ± 11.1	<0.001
Laboratory values						
WBC, × 109/L	11.5 ± 3.6	11.2 ± 3.9	0.663	6.3 ± 2.2	7.7 ± 2.6	0.055
Hemoglobin, g/dL	10.4 ± 2.4	11.0 ± 2.3	0.006	11.3 ± 2.4	10.9 ± 2.5	0.301
Platelet, × 109/L	253.6 ± 63.2	257.9 ± 77.5	0.703	232.7 ± 88.4	248.9 ± 93.8	0.320
Albumin, g/dL	2.9 ± 0.8	2.8 ± 0.8	0.726	3.5 ± 0.6	2.5 ± 0.7	0.492
Bilirubin, mmol/L	1.1 ± 0.4	1.2 ± 0.5	0.823	1.2 ± 0.5	1.2 ± 0.4	0.983
Bicarbonate, mEq/L	24.5 ± 4.5	23.8 ± 4.9	0.057	24.2 ± 3.6	23.6 ± 4.0	0.330
Blood urea nitrogen, mg/dL	22.6 ± 10.5	27.1 ± 12.6	0.006	14.3 ± 4.8	19.8 ± 6.7	0.013
Creatinine, mg/dL	1.3 ± 0.4	1.6 ± 0.5	0.002	0.9 ± 0.3	1.1 ± 0.4	0.002
Potassium, mmol/L	4.1 ± 0.7	4.1 ± 0.7	0.709	3.9 ± 0.5	4.1 ± 0.5	0.025
Sodium, mmol/L	137.2 ± 5.2	137.7 ± 5.2	0.191	139.1 ± 3.6	138.6 ± 4.1	0.340
Clinical outcomes						
Length of hospital stay, days	9.2 (6.0, 15.3)	11.4 (7.4, 19.2)	0.001	10.0 (6.1, 27.7)	12.0 (7.2, 39.2)	0.086
In-hospital death, n (%)	44 (7.7)	42 (22.7)	<0.001	11 (6.9)	11 (19.0)	0.009

WBC, White blood cell; SOFA, sequential organ failure assessment; OASIS, oxford acute severity of illness score; APSIII, acute physiology score III.

The primary outcome was AKI. The secondary outcome was in-hospital mortality.

### 2.4 Statistical analysis

All statistical analyses were performed using R software (version 4.2.2). Continuous variables with a normal distribution are presented as mean ± standard deviation, whereas non-normally distributed variables are expressed as median (interquartile range, IQR). Categorical variables are presented as frequencies and percentages. A two-sided p value< 0.05 was considered statistically significant.

To develop the AKI prediction model, univariable logistic regression was first performed to identify candidate predictors. Variables with statistical significance in the univariable analysis were then entered into a multivariable logistic regression model, and backward stepwise selection was used to identify independent predictors of AKI. A new risk score model was subsequently constructed based on the regression coefficients (*β* values) derived from the final multivariable logistic regression model. Model discrimination was assessed using receiver operating characteristic (ROC) curve analysis and compared with established scoring systems. Clinical utility was evaluated using decision curve analysis (DCA).

To assess the prognostic value of the newly developed AKI risk score for the secondary outcome, patients in both cohorts were categorized into high-risk and low-risk groups according to the predefined cutoff value of the risk score. Cox proportional hazards regression analysis was performed to evaluate the association between the risk score and in-hospital mortality. Survival time was defined as the interval from ICU admission to in-hospital death, and patients who survived to hospital discharge were censored at the date of discharge. Three sequential models were fitted: Model I was unadjusted; Model II was adjusted for age, sex, BMI, and comorbidities (congestive heart failure, hypertension, diabetes, and CKD); and Model III was further adjusted for laboratory variables, including HGB, PL, ALB, bilirubin, bicarbonate, BUN, creatinine, sodium, and potassium. Hazard ratios (HRs) and 95% confidence intervals (CIs) were reported.

Kaplan-Meier survival curves were generated for the high-risk and low-risk groups, and differences between groups were compared using the log-rank test. Additional analyses were performed to evaluate the predictive performance of the risk score for in-hospital mortality, including heatmaps of WBC-related markers, ROC curve analysis, and DCA.

Prespecified subgroup analyses were conducted to assess whether the predictive value of the new risk score model differed across clinically relevant patient subgroups. For AKI, subgroup-specific associations were assessed using logistic regression; for in-hospital mortality, Cox proportional hazards regression was used. Formal interaction was tested by incorporating a multiplicative interaction term between the risk group (high-risk vs. low-risk) and each subgroup variable, including age, sex, congestive heart failure, hypertension, diabetes, and CKD, into the corresponding multivariable model. The P value for interaction was derived using the likelihood ratio test (LRT), and the results were presented as forest plots. Waterfall plots were used to illustrate the distribution of the risk score and its relationship with different outcomes.

## 3 Results

### 3.1 General characteristics

A total of 981 critically ill patients with colorectal cancer were included in this study, comprising 764 patients from the eICU database in the training cohort and 217 patients from the Wuhan Union Hospital ICU in the validation cohort. Patients in each cohort were stratified according to the occurrence of AKI for comparison of baseline characteristics (**Table 1**).

In the training cohort, 185 of 764 patients (24.2%) developed AKI. Compared with patients without AKI, those with AKI had a higher prevalence of CKD (30/185 [16.2%] vs. 43/579 [7.4%], P< 0.001), higher disease severity scores, including SOFA (5.2 ± 1.5 vs. 2.9 ± 1.0), OASIS (29.5 ± 10.2 vs. 22.6 ± 9.7), and APS III (50.6 ± 19.6 vs. 41.8 ± 16.0) (all P< 0.001), a longer hospital stay (11.4 [7.4-19.2] vs. 9.2 [6.0-15.3] days, P = 0.001), and a higher in-hospital mortality rate (42/185 [22.7%] vs. 44/579 [7.7%], P< 0.001).

In the validation cohort, 58 of 217 patients (26.7%) developed AKI. Similarly, patients with AKI had a higher prevalence of CKD (15/58 [25.9%] vs. 17/159 [10.7%], P = 0.005), higher SOFA (4.8 ± 1.5 vs. 2.4 ± 1.0), OASIS (23.6 ± 6.1 vs. 19.6 ± 5.7), and APS III (39.9 ± 11.1 vs. 30.1 ± 7.1) scores (all P< 0.001), and a higher in-hospital mortality rate (11/58 [19.0%] vs. 11/159 [6.9%], P = 0.009). Length of hospital stay was numerically longer in the AKI group, although the difference was not statistically significant (12.0 [7.2-39.2] vs. 10.0 [6.1-27.7] days, P = 0.086).

### 3.2 Establishment of a new risk model

In the training cohort, 15 WBC-related indicators were screened by univariate logistic regression, of which 13 were significantly associated with AKI (all P< 0.05). These variables were subsequently entered into a multivariable logistic regression model. Four indicators remained independently associated with AKI: WBC-to-neutrophil ratio (WNR; OR 3.63, 95% CI 2.61-5.38, P< 0.001), Lg(WBC-to-eosinophil ratio [WER]; OR 2.03, 95% CI 1.25-3.30, P = 0.004), monocyte-to-lymphocyte ratio (MLR; OR 1.09, 95% CI 1.03-1.15, P = 0.002), and Lg(systemic immune-inflammation index [SII]; OR 3.45, 95% CI 2.54-4.16, P<0.001) (**Table 2**). Based on these four predictors, a novel risk score model for AKI in critically ill patients with advanced CRC was established as follows: risk score=1.271×WNR+0.709×Lg(WER)+0.085×MLR+1.225×Lg(SII).

**Table 2:** Logistic regression for WBC indicators and AKI (training set).

Variable	Univariate			Multivariate		
	*β*	OR (95%CI)	P	*β*	OR (95%CI)	P
WNR	1.802	6.06 (3.30-11.14)	<0.001	1.271	3.63 (2.61-5.38)	<0.001
WMR	0.001	1.00 (1.00-1.00)	<0.001	–	–	–
Lg (WMR)	0.619	1.86 (1.18-2.93)	0.008	0.555	1.74 (0.91-3.34)	0.094
WLR	0.067	1.07 (1.04-1.09)	<0.001	-0.028	0.97 (0.94-1.01)	0.077
WER	0.002	1.00 (1.00-1.00)	<0.001	–	–	–
Lg (WER)	0.646	1.91 (1.34-2.71)	<0.001	0.709	2.03 (1.25-3.30)	0.004
WBR	0.001	1.00 (1.00-1.00)	<0.001	–	–	–
Lg (WBR)	0.884	2.42 (1.59-3.69)	<0.001	0.299	1.35 (0.76-2.40)	0.308
WHR	-0.133	0.88 (0.71-1.09)	0.229	–	–	–
NLR	0.052	1.05 (0.93-1.19)	0.403	–	–	–
PLR	0.001	1.00 (1.00-1.00)	<0.001	–	–	–
Lg (PLR)	0.840	2.32 (1.40-3.83)	0.001	-0.121	0.89 (0.39-2.03)	0.774
MLR	0.129	1.14 (1.10-1.17)	<0.001	0.085	1.09 (1.03-1.15)	0.002
SII	0.001	1.00 (1.00-1.00)	<0.001	–	–	–
Lg (SII)	1.329	3.91 (2.39-6.61)	<0.001	1.225	3.45 (2.54-4.16)	<0.001

OR, Odds Ratio; 95% CI, 95% Confidence Interval; WBC, White Blood Count; AKI, Acute Kidney Injury; WNR, WBC to neutrophil ratio; WMR, WBC to monocyte ratio; WLR, WBC to lymphocyte ratio; WER, WBC to eosinophil ratio; WBR, WBC to basophil ratio; WHR, WBC to hemoglobin ratio; NLR, neutrophil-to-lymphocyte ratio; PLR, platelet-to-lymphocyte ratio; MLR, monocyte-to-lymphocyte ratio; SII, systemic immune-inflammation index.

### 3.3 Performance validation of the new AKI risk model

**Figure 2:**
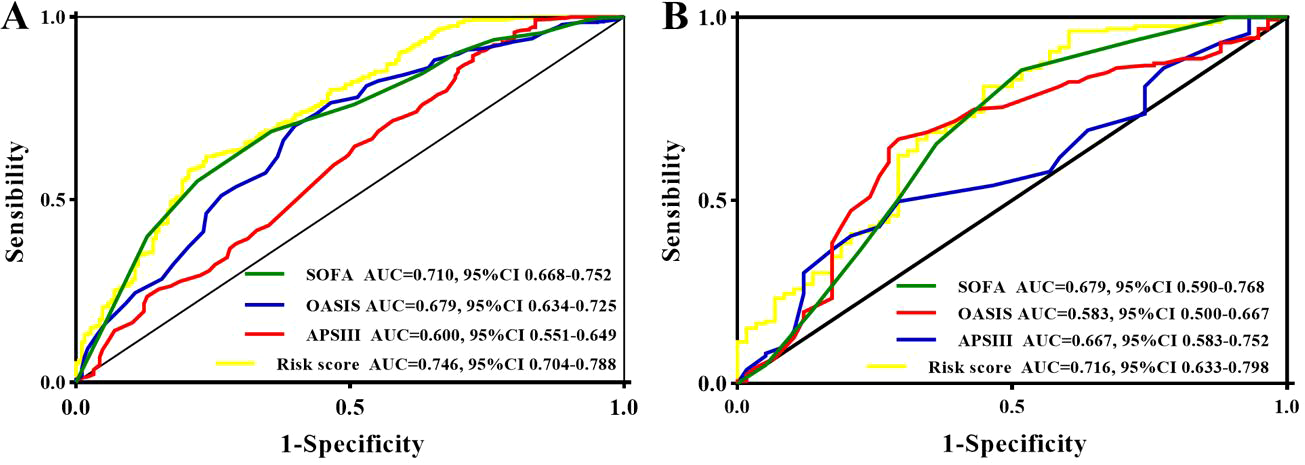
ROC curve analysis of SOFA, OASIS, APSIII, and the new AKI risk score model in the training set **(A)** and validation set **(B)**.

In the training set, ROC curve analysis (**Figure 2A**) showed that the AUC of the novel risk model was 0.746 (95% CI: 0.704-0.788). For predicting AKI, this model outperformed the SOFA (AUC = 0.710, 95% CI: 0.668-0.752), OASIS (AUC = 0.679, 95% CI: 0.634−0.725), and APS III (AUC = 0.600, 95%CI: 0.551−0.649) scoring systems. In the logistic regression of the AKI risk score, three sequentially adjusted models were constructed. Model I was unadjusted, whereas Models II and III were further adjusted for demographic/comorbidity variables and laboratory variables, respectively. When the risk score was analyzed as a continuous variable, the ORs were 2.49 (95 % CI: 1.39−4.45, P< 0.001) for Model I, 2.21 (95 % CI: 1.20−4.10, P = 0.001) for Model II, and 2.07 (95 % CI: 1.11−3.86, P = 0.011) for Model III. Using a cut-off value of 7.80, patients were stratified into high-risk and low-risk groups. When examined as a categorical variable, the high-risk group, compared with the low-risk group, had ORs of 3.04 (95% CI: 1.56-5.91, P< 0.001), 2.77 (95% CI: 1.47-5.22, P = 0.002), and 2.61 (95% CI: 1.44-4.73, P = 0.002) in Models I, II, and III, respectively (**Table 3**).

In the validation set, ROC curve analysis outcomes (**Figure 2B**) demonstrated that the AUC of the new risk model was 0.716 (95% CI: 0.633-0.798). In predicting AKI, this model also surpassed the SOFA (AUC = 0.679, 95% CI: 0.590-0.768), OASIS (AUC = 0.583, 95% CI: 0.500-0.667), and APS III (AUC = 0.667, 95% CI: 0.583-0.752) assessment tools. The ORs values for the risk score as a continuous variable in Models I, II, and III were 2.65 (95% CI: 1.63-4.31, P< 0.001), 2.20 (95% CI: 1.45-3.34, P< 0.001), and 2.09 (95% CI: 1.40-3.13, P< 0.001), respectively. Analysis of categorical variables showed that the ORs for the high−risk group across the three models were 5.18 (95% CI: 2.24-11.96, P< 0.001), 4.86 (95% CI: 2.13-10.18, P< 0.001), and 4.50 (95% CI: 2.03–9.98, P< 0.001) in Models I, II, and III, respectively (**Table 3**).

The results show that the AKI risk score model showed stable predictive performance in both the training and validation sets and remained significantly associated with AKI after multivariable adjustment.

**Figure 3:**
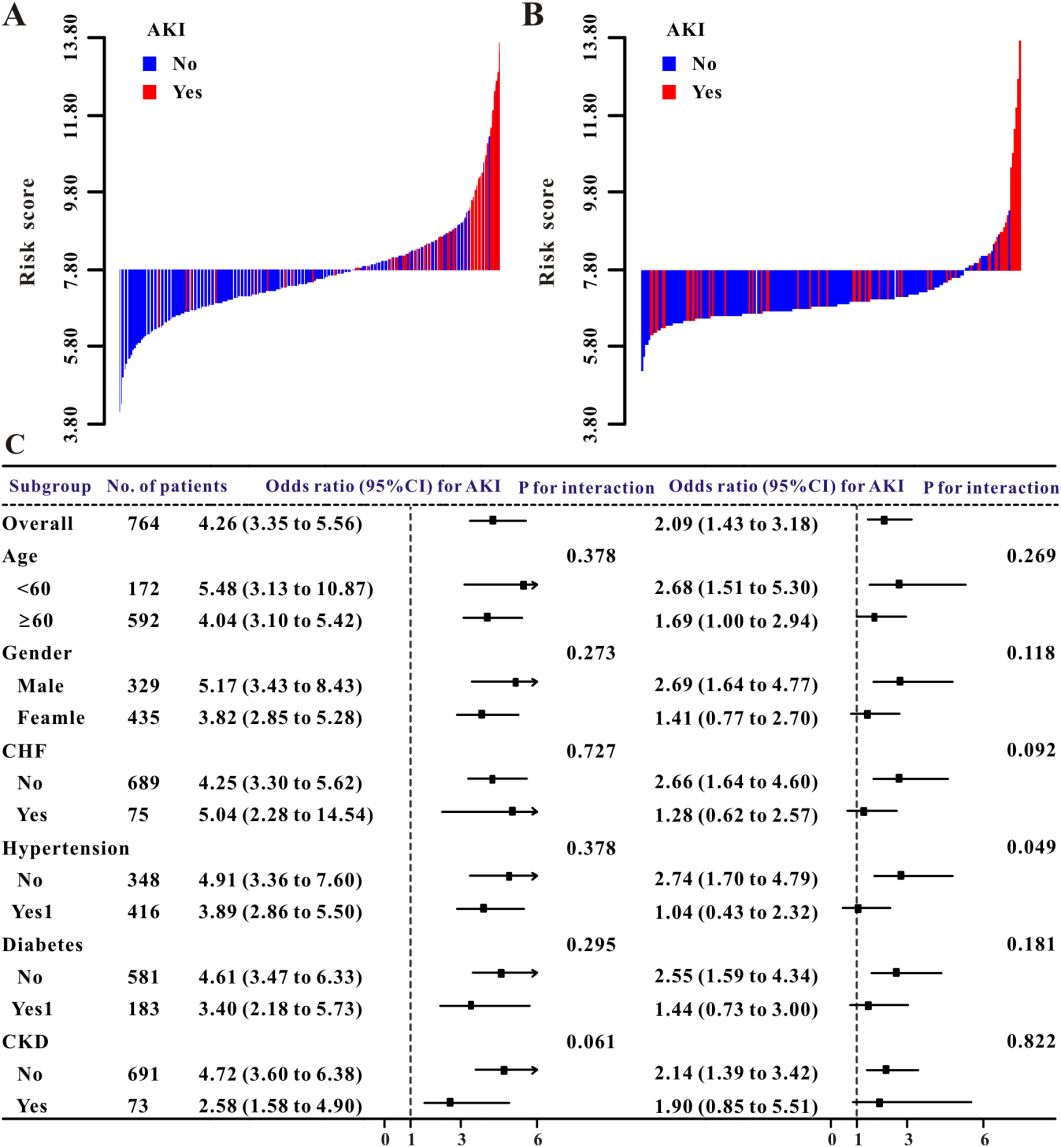
AKI risk model assessment. **(A)** Training set waterfall plot (high- vs. low-risk groups). **(B)** Validation set waterfall plot (high- vs. low-risk groups). **(C)** Subgroup analysis forest plot (training and validation sets).

**Figure 4:**
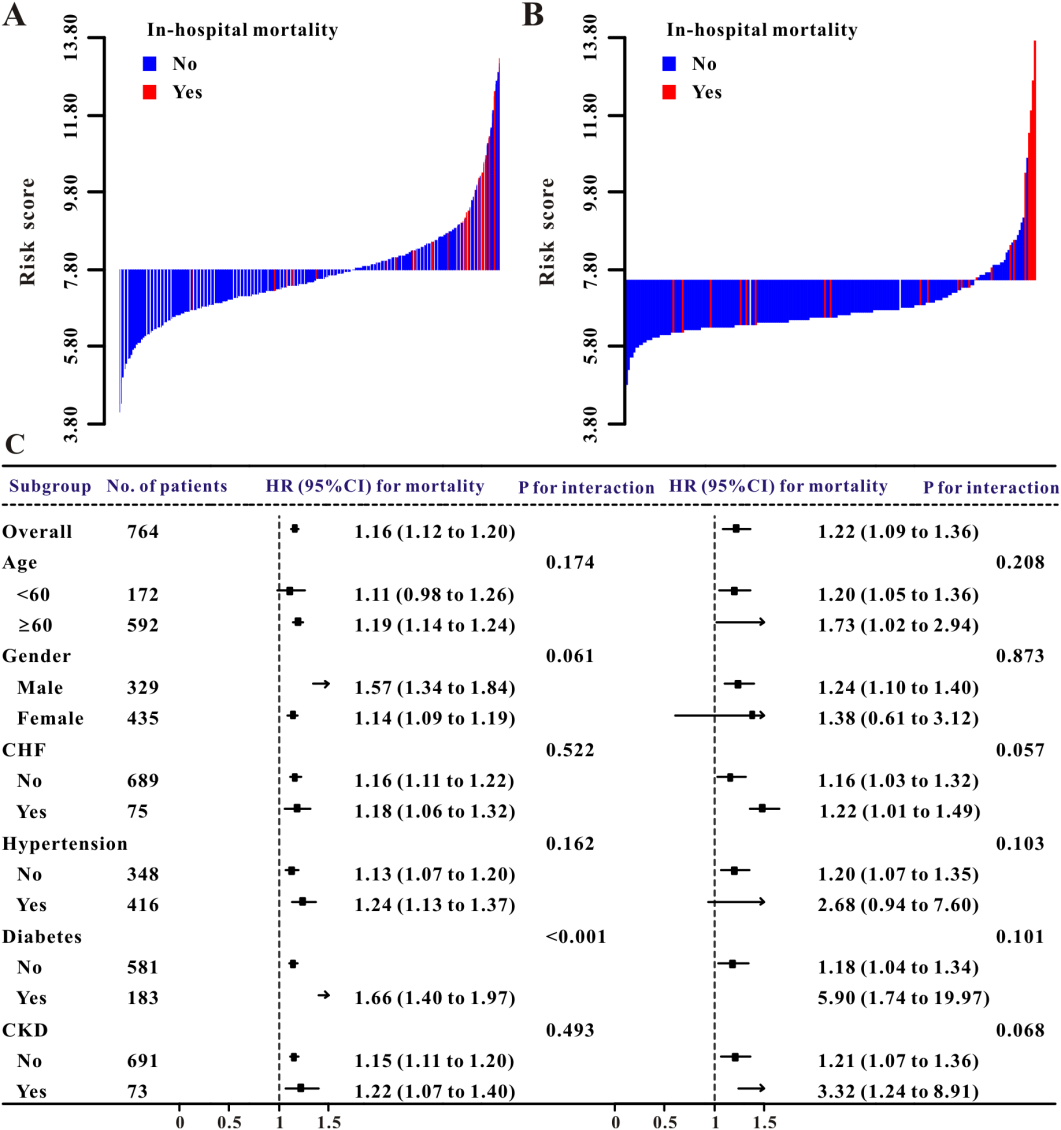
In-hospital mortality risk model assessment. **(A)** Training set waterfall plot (high- vs. low-risk groups). **(B)** Validation set waterfall plot (high- vs. low-risk groups). **(C)** Subgroup analysis forest plot (training and validation sets).

In the training and validation cohorts, the waterfall plot results (**Figures 3A, B**) demonstrated an obvious association between acute renal injury and higher risk scores. Elevated risk scores were directly linked to in−hospital mortality in both the training and validation groups, as shown by the waterfall plots (**Figures 4A, B**).

To evaluate the robustness and generalizability of the new risk score mode, we assessed its predictive performance across key patient subgroups defined by age, sex, and major comorbidities. Forest plots (**Figure 3C**) showed no significant interaction across key subgroups. The predictive ability of the risk score remained consistent in patients aged ≥ 60 years (OR 4.04, 95% CI: 3.10-5.42) and< 60 years (OR 5.48, 95% CI: 3.13-10.87; P for interaction = 0.378), as well as in male (OR 5.17, 95% CI: 3.43-8.43) and female patients (OR 3.82, 95% CI: 2.85-5.28; P for interaction = 0.62). In patient subgroups with or without comorbid chronic heart failure, hypertension, diabetes or CKD, no significant heterogeneity in predictive power was found (all P for interaction > 0.05), and the predictive ability of the new risk score mode showed consistency. In the validation set, point estimates and confidence intervals overlapped partially between patients with CKD (OR 1.90, 95% CI: 0.85-5.51) and non-CKD patients (OR 2.14, 95% CI: 1.39-3.42), and the interaction was not significant (P for interaction = 0.822).

Subgroup analysis of the in-hospital mortality risk score was presented using forest plots (**Figure 4C**). The results indicated that the score demonstrated robust and consistent predictive ability across most predefined subgroups. The association between the score and in-hospital mortality was stable across patients of different ages and genders. The point estimates for the predictive power of the score were similar in patients aged ≥ 60 years (HR 1.11, 95% CI: 0.98-1.26) and those aged< 60 years (HR 1.19, 95% CI: 1.14-1.26), and the confidence intervals overlapped (P for interaction = 0.174). No significant difference in predictive power was observed between male and female patients (P for interaction = 0.061).Across subgroups stratified by major comorbidities, including congestive heart failure, hypertension, diabetes, and CKD, no significant heterogeneity was detected.

**Table 3:** Logistic regression analysis of the risk score for AKI.

Methods	Model I		Model II		Model III	
	OR (95%CI)	P	OR (95%CI)	P	OR (95%CI)	P
In the training set						
Risk score (continues)	2.49 (1.39-4.45)	<0.001	2.21 (1.20-4.10)	0.001	2.07 (1.11-3.86)	0.011
Risk score (category)	–	–	–	–	–	–
Low risk	Reference	–	Reference	–	Reference	–
High risk	3.04 (1.56-5.91)	<0.001	2.77 (1.47-5.22)	0.002	2.61 (1.44-4.73)	0.002
In the validation set						
Risk score (continues)	2.65 (1.63-4.31)	<0.001	2.20 (1.45-3.34)	<0.001	2.09 (1.40-3.13)	<0.001
Risk score (category)	–	–	–	–	–	–
Low risk	Reference	–	Reference	–	Reference	–
High risk	5.18 (2.24-11.96)	<0.001	4.86(2.13-10.18)	<0.001	4.50 (2.03-9.98)	<0.001

AKI, acute kidney injury; OR, odds ratio; 95%CI, 95% confidence index. Model I was unadjusted. Model II adjusted for model I plus age, gender, body mass index, and comorbidities. Model III adjusted for model II plus laboratory results, including Hemoglobin, Platelet, Albumin, Bilirubin, Bicarbonate, Blood urea nitrogen, Creatinine, Potassium.

### 3.4 Validation of the novel model for in-hospital mortality

**Figure 5:**
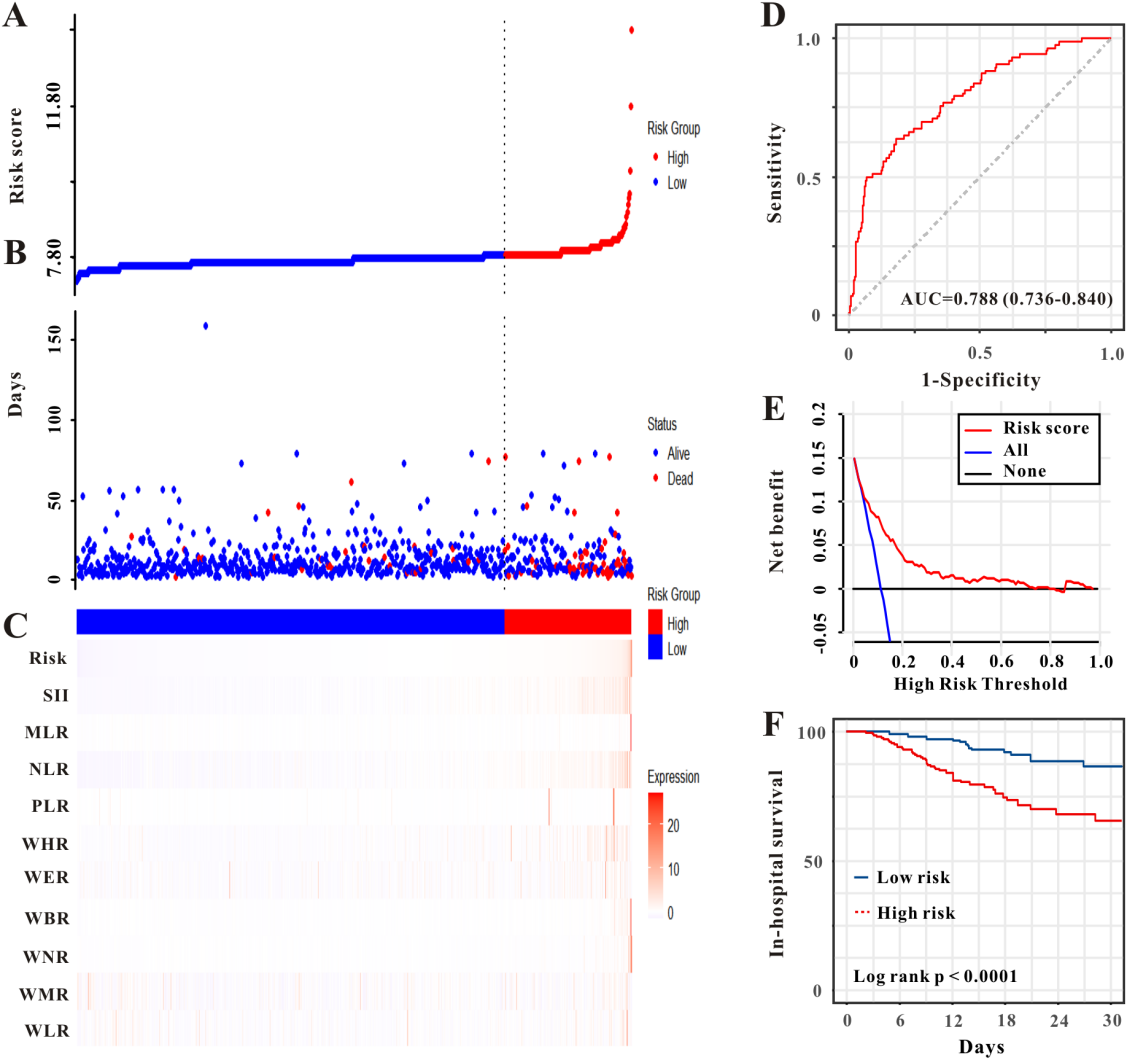
Evaluation of the AKI-derived risk score for predicting in-hospital mortality in patients with CRC (CRC) in the training set. **(A)** Stratification of patients into high- and low-risk groups based on the previously established AKI risk score. **(B)** Association between survival time and prognosis in the two risk groups. **(C)** Heatmap of WBC-based markers across the risk groups. **(D)** ROC curve assessing the model's discrimination for in-hospital mortality. **(E)** DCA evaluates the clinical utility of the model. **(F)** Kaplan-Meier curves comparing in-hospital mortality between the risk groups.

**Figure 6:**
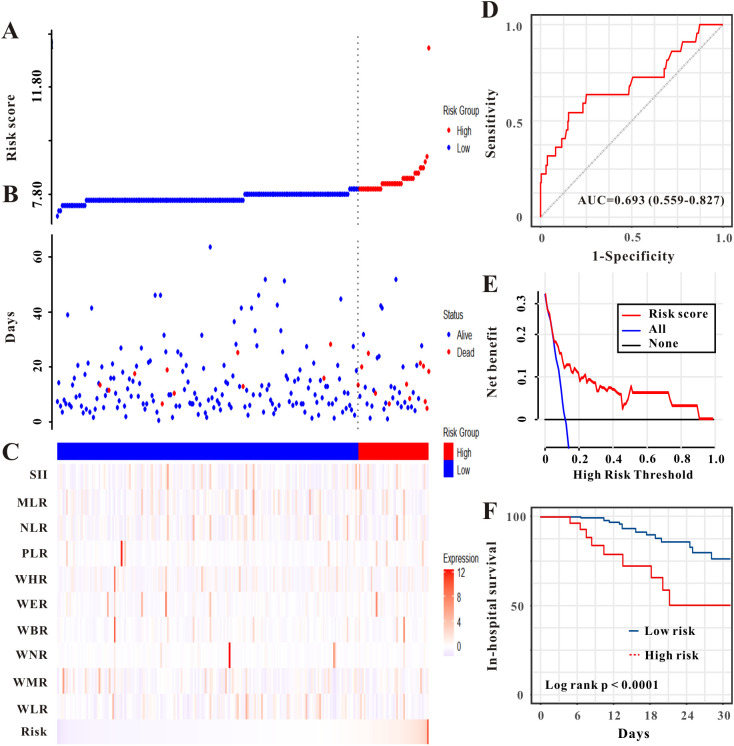
Evaluation of the AKI-derived risk score for predicting in-hospital mortality in patients with CRC (CRC) in the validation set. **(A)** Stratification of patients into high- and low-risk groups based on the previously established AKI risk score. **(B)** Association between survival time and prognosis in the two risk groups. **(C)** Heatmap of WBC-based markers across the risk groups. **(D)** ROC curve assessing the model's discrimination for in-hospital mortality. **(E)** DCA evaluates the clinical utility of the model. **(F)** Kaplan-Meier curves comparing in-hospital mortality between the risk groups.

We evaluated the prognostic utility of the novel risk score model for predicting in-hospital mortality in critically ill patients with advanced CRC. In the training set, as shown in **Figures 5A–C**, higher risk scores were associated with a greater likelihood of in-hospital death. The risk score for predicting in-hospital mortality showed an AUC of 0.788 (95% CI: 0.736-0.840) in the ROC analysis (**Figure 5D**). The decision curve analysis indicated a favorable net benefit across various threshold probabilities, thereby validating the clinical applicability of the model (**Figure 5E**). Furthermore, the Kaplan-Meier analysis illustrated lower in-hospital survival rates for the high-risk cohort in comparison to the low-risk cohort (log-rank P< 0.0001, **Figure 5F**). In the validation set, heatmaps (**Figures 6A–C**) showed that higher risk scores were associated with a higher likelihood of in-hospital death. For in-hospital mortality prediction, the AUC of the risk score was 0.693 (95% CI: 0.559-0.827), indicating acceptable discriminatory ability (**Figure 6D**). Decision curve analysis showed that the use of the risk model provided a net benefit for predicting in-hospital mortality across a range of threshold probabilities (**Figure 6E**). Kaplan-Meier curves demonstrated a significant divergence in in-hospital survival, with the high-risk cohort showing markedly poorer outcomes (log-rank P< 0.0001, **Figure 6F**).

To evaluate the predictive performance and robustness of the risk score, this study examined its association with in-hospital mortality using Cox proportional hazards models with varying degrees of adjustment in both training and validation sets. The HR for the risk score as a continuous variable was 1.16 (95% CI: 1.12-1.20, P< 0.001) for Model I, 1.15 (95% CI: 1.11-1.20, P< 0.001) for Model II, and 1.10 (95% CI: 1.07-1.18, P< 0.001) for Model III, in the training set. When analyzed by category, the high-risk group compared to the low-risk group had HRs of 3.34 (95% CI: 2.08-5.51, P< 0.001), 3.12 (95% CI: 1.92-5.36, P< 0.001), and 2.81 (95% CI: 1.70-4.66, P< 0.001) for the three models, respectively. In the validation set, the HR for the continuous risk score was 1.19 (95% CI: 1.09-1.36, P< 0.001) for Model I, 1.24 (95% CI: 1.09-1.42, P = 0.001) for Model II, and 1.18 (95% CI: 1.01-1.38, P = 0.046) for Model III. The high-risk group had HRs of 3.84 (95% CI: 1.64-9.00, P = 0.002), 4.17 (95% CI: 1.53-11.38, P = 0.005), and 3.61 (95% CI: 1.03-12.62, P = 0.044) for the three models, respectively. Details are shown in **Table 4**. The results demonstrate that this risk score had significant predictive ability for in-hospital mortality risk in both the training and validation sets, and remained statistically significant after multivariate adjustment, indicating good independent predictive power and clinical applicability.

**Table 4:** Multivariate COX regression analysis of risk score for in-hospital mortality.

Methods	Model I		Model II		Model III	
	HR (95%CI)	P	HR (95%CI)	P	HR (95%CI)	P
In the training set						
Risk score (continues)	1.16 (1.12-1.20)	<0.001	1.15 (1.11-1.20)	<0.001	1.10 (1.07-1.18)	<0.001
Risk score (category)	–	–	–	–	–	–
Low risk	Reference	–	Reference	–	Reference	–
High risk	3.34 (2.08-5.51)	<0.001	3.12 (1.92-5.36)	<0.001	2.81 (1.70-4.66)	<0.001
In the validation set						
Risk score (continues)	1.19 (1.09-1.36)	<0.001	1.24 (1.09-1.42)	0.001	1.18 (1.01-1.38)	0.046
Risk score (category)	–	–	–	–	–	–
Low risk	Reference	–	Reference	–	Reference	–
High risk	3.84 (1.64-9.00)	0.002	4.17(1.53-11.38)	0.005	3.61 (1.03-12.62)	0.044

AKI, acute kidney injury; HR, hazard ratio; 95%CI, 95% confidence index. Model I was unadjusted. Model II adjusted for model I plus age, gender, body mass index, and comorbidities. Model III adjusted for model II plus laboratory results, including Hemoglobin, Platelet, Albumin, Bilirubin, Bicarbonate, Blood urea nitrogen, Creatinine, Potassium.

## 4 Discussion

WBC−associated markers have so far not been employed in research to evaluate AKI risk and the prognosis of CRC patients, especially within the intensive care unit. This study specifically targeted the specific population of critically ill patients with advanced CRC in the complex ICU environment for AKI risk assessment.

Univariate analysis demonstrated that patients with AKI presented with a more severe clinical profile, reflected in significantly higher baseline disease severity scores and poorer underlying renal function, as evidenced by elevated BUN, elevated creatinine, and a greater prevalence of CKD. Ultimately, AKI patients faced longer hospital stays and significantly increased in-hospital mortality. In the multivariable model, four parameters—WNR, WER, MLR, and SII—retained significant independent associations with AKI risk in individuals with CRC. Using these indicators, a new risk model for AKI in critically ill CRC patients was constructed. This model can more accurately predict the occurrence of AKI in patients with advanced CRC, which helps doctors make earlier judgments about the patient’s condition and take preventive and therapeutic measures, thereby improving the prognosis of the patients. Unlike most studies that only perform internal validation, this study used an independent external dataset for rigorous validation of the mode.

The evaluation and validation results of the new risk model both demonstrated that the it exhibits strong predictive accuracy and reliability. Decision curve analysis results also showed that this prediction model can achieve good clinical net benefit within the threshold range and has good generalization ability. Moreover, the model demonstrated strong performance in assessing both the risk of AKI occurrence and in-hospital mortality in CRC patients. Patients can be classified into high-risk and low-risk categories according to their risk scores. This enables clinicians to monitor high−risk patients more closely and implement interventions such as avoiding nephrotoxic drugs and promptly adjusting hemodynamics. This risk score is also a strong predictor of in-hospital mortality, with a high-risk score indicating poorer in-hospital survival, aiding doctors in assessing the overall severity of a patient’s condition and facilitating more comprehensive communication with relatives.

AKI is one of the important complications of CRC, and the management of AKI risk in CRC patients is a key focus in the clinical diagnosis and treatment process. The occurrence of AKI varies depending on the treatment modality. The incidence of AKI in metastatic CRC patients receiving immunotherapy is 3.4% (18). The incidence of AKI in patients having major surgery might reach 11% (19). Renal damage occurred in 99 (22.4%) of the 442 patients who underwent ileostomy following surgery for a colorectal tumor (20). A study by Kirsty Andresen et al. showed that during a one-year follow-up per 1000 CRC survivors, 29.3 were expected to newly develop AKI (21). The findings from this research indicated that data collected from Wuhan Union Hospital revealed an incidence rate of AKI among CRC patients who were admitted to the ICU to be around 27.1%. Furthermore, an examination of the eICU database demonstrated that the occurrence of AKI in critically ill CRC patients was approximately 24.2%. Comparisons of multiple data sources indicate that the risk of AKI in critically ill CRC patients exceeds that of other patients. Currently, the incidence of AKI among patients with CRC is attributed to multiple factors.

CRC patients are susceptible to complications such as gastrointestinal bleeding, nausea and vomiting, and intestinal obstruction, leading to decreased effective circulating blood volume and resulting in renal hypo-perfusion. In addition, chemotherapy agents, targeted therapies, immune checkpoint inhibitors, and similar treatments have possible nephrotoxic effects, and there is also a risk of radiation−induced kidney injury (22). CRC patients undergoing surgery may experience surgical stress, intraoperative blood loss, improper fluid management, and other issues leading to renal ischemia. Additionally, complications such as infection and sepsis can induce systemic inflammatory response and renal vasoconstriction. A meta-analysis identified several risk factors for AKI in individuals diagnosed with CRC, including male sex, older age, BMI > 25 kg/m², diabetes mellitus, hypertension, CKD, hypoalbuminemia, and surgical-related variables (23). These findings are largely in agreement with the outcomes observed in the present study. The superposition of these factors collectively leads to the acute deterioration of glomerular filtration function in CRC patients.

Previously, several scholars have developed models to predict AKI in cancer patients using various methodological approaches. Scanlon et al. (24) developed a machine learning model that demonstrated significant advantages in predictive performance (AUROC = 0.881). However, the clinical application of such models still faces multiple challenges. First, machine learning algorithms such as random forest typically require the integration of a large number of clinical variables and rely on complete electronic medical record systems and backend computing resources, making them difficult to implement in real time in resource-limited ICU settings. Second, the lack of transparency in how these models arrive at their predictions prevents clinicians from intuitively understanding which indicators and with what weights the model derives a patient’s risk probability of developing AKI. In the high-risk environment of the ICU, clinicians often need a clear grasp of the decision-making logic before they can trust and adopt the model’s recommendations. In contrast, the linear scoring model developed in this study requires only four WBCl-related ratios, can be quickly calculated at the bedside, and has clearly identifiable variable weights, allowing clinicians to intuitively understand the contribution of each indicator to the risk score. This makes our model better suited to the real-world needs of the ICU, enhancing clinical practicality and potential for widespread adoption while maintaining acceptable predictive performance.

In the specific context of CRC surgery, Liu Li et al. (25) identified preoperative hypertension, preoperative anemia, intraoperative crystalloid infusion volume, intraoperative minimum mean arterial pressure, and moderate to severe postoperative hemoglobin decline as independent risk factors, constructing a model with an AUC of 0.776. Although this model is well-suited for the postoperative setting, it does not cover the broader population of critically ill patients with advanced CRC admitted to the ICU for various non-surgical reasons.

More recently, studies have begun exploring the application of WBC-related indicators for AKI prediction in other critically ill populations. Peng et al. (26) developed a risk model for AKI in critically ill patients with hepatocellular carcinoma based on WNR, white blood cell count to mean platelet volume ratio (WMR),white blood cell to hemoglobin ratio (WHR), and platelet-to-lymphocyte ratio (PLR), demonstrating good predictive performance in the training set, internal validation set, and external validation set. Similarly, Liu et al. (27) established an AKI prediction model for patients with ischemic stroke admitted to the ICU based on white blood cell to lymphocyte ratio (WLR),white blood cell to low-density lipoprotein cholesterol ratio (WBR), WHR, and neutrophil-to-lymphocyte ratio (NLR), which showed robust predictive ability for both AKI and in-hospital mortality. These studies collectively support the broad applicability of white blood cell-related indicators as practical and accessible predictors of AKI in critically ill populations.

Compared with the existing models described above, the present study offers several advantages. First, unlike the machine learning approach employed by Scanlon et al. (24), our model is a simple linear equation composed of four easily obtainable WBC-related ratios (WNR, WER, MLR, SII), which can be conveniently used and interpreted at the bedside without requiring complex computational infrastructure. Second, in contrast to the model developed by Liu Li et al. (25) for the postoperative population, our study focuses on the broader population of critically ill patients with advanced CRC admitted to the ICU, covering a more heterogeneous group. Third, a major strength of this study lies in its rigorous external validation using an independent cohort from Wuhan Union Hospital, where the model maintained stable predictive performance (AUC = 0.716), strongly supporting its generalizability. Furthermore, our model demonstrates a unique dual functionality: beyond predicting AKI occurrence, it also shows robust predictive value for in-hospital mortality, with an AUC of 0.788 in the training set and 0.693 in the validation set, and remained statistically significant after multivariate adjustment. The ability to achieve two purposes with a single score makes this model highly practical in the ICU, allowing clinicians to identify both patients at high risk for AKI and those with a poorer overall prognosis. The new model developed in this study demonstrates good discrimination, calibration, and clinical utility, and remains stable across different subgroup populations, providing a practical tool for early AKI risk stratification in critically ill patients with advanced CRC. Meanwhile, compared to existing scoring systems such as SOFA, OASIS, and APSIII, the new risk score model showed better performance.

## 5 Conclusions

This study utilized WBC-related indicators, including WNR, WER, MLR, and SII, to establish a novel prediction model for AKI in critically ill patients with advanced CRC. For critically ill patients with advanced CRC, the model showed predictive value for both AKI and in-hospital mortality, and may help clinicians identify high-risk patients early, optimize monitoring intensity, and implement timely preventive and therapeutic interventions.

## 6 Shortcomings and prospects

The model constructed in this study demonstrated good performance, but there are still limitations. First, the retrospective design of this study entails inherent limitations, and its conclusions are susceptible to confounding factors and selection bias. Second, the data for this study came from the public database eICU and the Wuhan Union Hospital ICU data; differences in data collection methods and quality between the two may have some impact on the results. Third, due to limitations in database information, we were unable to obtain accurate tumor staging and key clinical details closely related to AKI occurrence, such as the specific nephrotoxic drugs used by patients, precise volume status, and detailed hemodynamic monitoring data. The lack of this information may limit the comprehensiveness and precision of the model. Fourth, this study excluded patients with a hospital stay of less than 48 hours. Although this was necessary to ensure the completeness and consistency of the data, it may have introduced survivor bias and potentially underestimated the actual risk and impact of AKI during its early stages. Future prospective multicenter studies in broader and more diverse populations are needed to further validate the model, incorporate additional clinically relevant variables, explore the value of dynamic monitoring of WBC-related indicators, and determine whether application of this model in clinical practice can improve early risk stratification, optimize management, and ultimately improve outcomes in critically ill patients with advanced CRC.

Future prospective multicenter studies in broader and more diverse populations are needed to further validate the model, incorporate additional clinically relevant variables, explore the value of dynamic monitoring of WBC-related indicators, and determine whether application of this model in clinical practice can improve early risk stratification, optimize management, and ultimately improve outcomes in critically ill patients with advanced CRC.

## Data Availability

The raw data supporting the conclusions of this article will be made available by the authors, without undue reservation.
