# Supplementary material for: Development of a prediction model for acute kidney injury in critically ill patients with advanced colorectal cancer based on white blood cell-related indicators
Source: Front Oncol. 2026 Apr 27;16:1768330. doi: 10.3389/fonc.2026.1768330 (PMC13158105; doi:10.3389/fonc.2026.1768330)
Supplement: Supplementary file 1 [file Table1.docx]

Supplementary Table 1

| **Abbreviation** | **Full Name** | **Definition and Formula** |
| --- | --- | --- |
| WNR | White blood cell-to-Neutrophil Ratio | Ratio of white blood cell count to neutrophil count (WBC / Neutrophil count) |
| WMR | White blood cell-to-Monocyte Ratio | Ratio of white blood cell count to monocyte count (WBC / Monocyte count) |
| Lg (WMR) | Base-10 logarithm of WMR | The base-10 logarithmic transformation of the WMR value (Log10 WMR) |
| WLR | White blood cell-to-Lymphocyte Ratio | Ratio of white blood cell count to lymphocyte count (WBC / Lymphocyte count) |
| WER | White blood cell-to-Eosinophil Ratio | Ratio of white blood cell count to eosinophil count (WBC / Eosinophil count) |
| Lg (WER) | Base-10 logarithm of WER | The base-10 logarithmic transformation of the WER value (Log10 WER) |
| WBR | White blood cell-to-Basophil Ratio | Ratio of white blood cell count to basophil count (WBC / Basophil count) |
| Lg (WBR) | Base-10 logarithm of WBR | The base-10 logarithmic transformation of the WBR value (Log10 WBR) |
| WHR | White blood cell-to-Hemoglobin Ratio | Ratio of white blood cell count to hemoglobin level (WBC / Hemoglobin) |
| NLR | Neutrophil-to-Lymphocyte Ratio | Ratio of neutrophil count to lymphocyte count (Neutrophil / Lymphocyte count) |
| PLR | Platelet-to-Lymphocyte Ratio | Ratio of platelet count to lymphocyte count (Platelet / Lymphocyte count) |
| Lg (PLR) | Base-10 logarithm of PLR | The base-10 logarithmic transformation of the PLR value (Log10 PLR) |
| MLR | Monocyte-to-Lymphocyte Ratio | Ratio of monocyte count to lymphocyte count (Monocyte / Lymphocyte count) |
| SII | Systemic Immune-Inflammation Index | Calculated as Platelet count × Neutrophil count / Lymphocyte count |
| Lg (SII) | Base-10 logarithm of SII | The base-10 logarithmic transformation of the SII value (Log10 SII) |
